# Thrombotic Microangiopathy and Multiple Organ Failure in Scleroderma Renal Crisis: A Case Report

**DOI:** 10.7759/cureus.44322

**Published:** 2023-08-29

**Authors:** Lorenzo Aterini, Marco Gallo, Barbara Vadalà, Stefano Aterini

**Affiliations:** 1 Nephrology, AOU (Azienda Ospedaliera Universitaria) Meyer Children Hospital, School of Human Health Sciences at University of Firenze, Firenze, ITA; 2 Hemodialysis Centre, Istituto Fiorentino di Cura e Assistenza (IFCA), Firenze, ITA

**Keywords:** microangiopathic hemolytic anemia (maha), multiple organ failure, plasmic score, thrombotic microangiopathy, acute kidney injury, scleroderma renal crisis, systemic sclerosis, scleroderma

## Abstract

This case report can be considered a rare occurrence of scleroderma renal crisis (SRC) presenting with a severe clinical course and multiple organ failure. A patient diagnosed with systemic sclerosis four years earlier was admitted to the hospital because of severe malignant systolic-diastolic arterial hypertension and acute kidney injury (AKI). Exacerbating disease suggested thrombotic microangiopathy (TMA) and the PLASMIC (Platelet count; combined hemoLysis variable; absence of Active cancer; absence of Stem-cell or solid-organ transplant; MCV; INR; Creatinine) score was used in the differential diagnosis. Despite the timely initiation of therapy with ACE inhibitors (ACE-I), the progressive renal failure required hemodialysis treatment, but renal function never recovered. Disease duration, skin involvement, and previous specific pharmacological therapy represented multiple risk factors that determined a clinical course complicated by pericardial tamponade with acute heart failure, acute pancreatitis, and ischemic stroke, with fatal evolution. These complications presented a challenging clinical sequence of events requiring an interdisciplinary course of action. Timely ascertainment of the SRC is important given the possible severe organ involvement as well as mortality. A diagnosis of scleroderma renal crisis should be considered in cases of acute kidney injury associated with known risk factors. Early treatment and collaboration between rheumatology and renal physicians can improve patient outcomes.

## Introduction

Scleroderma (or systemic sclerosis) is an autoimmune systemic disease characterized by thickening and progressive fibrosis of the skin, with involvement of vessels and visceral organs including the gastrointestinal tract, lungs, heart, and kidneys [1]. The disease is highly variable in its clinical presentations and degree of organ involvement.

Scleroderma renal crisis (SRC), characterized by acute onset of renal failure associated with severe hypertension, sometimes with a malignant feature, is the most alarming form of renal involvement, being one of the few emergencies in clinical rheumatology [2]. It involves approximately 10% of affected patients [3]. In some cases, SRC clinical presentation is similar to that of thrombotic microangiopathy (TMA) making a differential diagnosis necessary, mainly for the different therapeutic options [1,4-7]. The PLASMIC score was developed to distinguish between TMA patients with and without metalloprotease ADAMTS13 activity level ≤10%. The name “PLASMIC” refers to the score’s seven components: Platelet count; combined hemoLysis variable; absence of Active cancer; absence of Stem-cell or solid-organ transplant; MCV; INR; Creatinine [8]. The patient described in this case report can be considered a rare occurrence of SRC presenting with clinical and laboratory data suggesting a secondary thrombotic microangiopathy complicated by a severe clinical course with multiple organ failure.

## Case presentation

The medical history of a 65-year-old patient centered around the diagnosis of systemic sclerosis (SS) four years earlier. Presenting symptoms included Raynaud's phenomenon in the fingertips, diffuse telangiectasias, and progressive skin thickening of the limbs. Autoimmune workup was positive for antinuclear antibodies 1:1,280 and anti-centromere antibody level was > 240 IU/ml. Antibodies anti-Topoisomerase I (anti-Scl-70) were positive, while antibodies anti-RNA Polymerase III, anti-dsDNA, and rheumatoid factor were negative. Serum complement was normal: C3 161 mg/dl (90-180 mg/dl) and C4 29 mg/dl (10-40 mg/dl). Nailfold capillaroscopy was consistent with the active scleroderma pattern. Diffuse bilateral interstitial pulmonary fibrosis was observed on thoracic computed tomography. An echocardiogram revealed left ventricular dilatation and diastolic dysfunction with an ejection fraction of 48%. The patient was regularly examined at the local specialist outpatient clinic and, at first, treated with immunoglobulin and mycophenolate mofetil (2 g/day). Because of interstitial lung disease progression, therapy was switched to monthly IV cyclophosphamide, interrupted after four infusions due to the lack of any clinical improvement. Treatment was then reassessed by administering glucocorticoids (methylprednisolone 1000 mg/day for three days and later, prednisone with a gradually tapered dose) and rituximab 375 mg/m^2^ at two-week intervals. In spite of the new therapeutic regimen, the patient suffered a progression of skin involvement, with supple skin, diffusely erythematous with intense itching, presence of evening fever, and worsening of exertional dyspnoea. Chest CT angiography excluded cardiac as well as pleuro-parenchymal modifications. Renal evaluation found no evidence of parenchymal or vascular abnormalities, with unremarkable urinary sediment. A few days later, the patient experienced headache, dyspnoea at rest, lumbar pain, and a progressive worsening of the general condition, with loss of functional autonomy. The general practitioner found high blood pressure (180/110 mmHg); HR 110 bpm; temperature 37.7°C; Sat O_2_ 96% in air. Blood tests showed elevated creatinine (3.8 mg/dl) and markedly reduced haemoglobin (8.1 g/dl), requiring hospital admission with a diagnosis of acute kidney injury (AKI). The initial admission creatinine level was 4.6 mg/dl, with findings of proteinuria, active urine sediment, and microhematuria in the urine analysis. The blood count analysis revealed severe normocytic anemia, neutrophilic leukocytosis, and thrombocytopenia. Peripheral blood smear findings showed schistocytes at 3%. Elevated inflammatory markers yielded high results (Table 1).

**Table 1:** Patient’s laboratory values RBC: red blood cells; PLT: platelet values; eGFR: estimated glomerular filtration rate; CRP C-reactive protein; BP: blood pressure

	60 days before	20 days before	At admission	Normal range
Leukocytes 10^3^/mmc	7.53	17.10	20.0	4.0-11.0
Neutrophils (%)	61.5	81.4	86	40-70
RBC 10^6^/mmc	4.28	4.17	2.77	4.2-6.1
Hemoglobin g/dl	11.0	11.4	7.5	11-14
PLT 10^3^/mmc	374	348	247	140-440
Creatinine mg/dl	0.45	0.63	4.6	0.6-1.0
eGFR ml/min/1,73 mq	> 90	>90	9	>90
Na mEq/l	139	138	131	136-146
K mEq/l	4.1	4.1	4.9	3.5-5.0
Total protein g/dl	7.1	6.0	4.9	6.0-8.0
Albumin g/dl	3,14	3.01	2.6	3.5-5.2
Proteinuria mg/dl	Negative	Negative	50 mg/dl	Negative
CRP mg/L	2.04	5.69	200	<5
BP mmHg	125/75	115/65	160/105	

Marked reduction of hemoglobin, 30% reduction of circulating platelets compared to previous values, reticulocytosis, negative direct Coombs test, and elevated serum LDH suggested hemolysis, even though haptoglobin and indirect bilirubin were within normal values (Table 2).

**Table 2:** Laboratory variables for TMA evaluation TMA: thrombotic microangiopathy; MCV: mean corpuscular volume; PLT: platelet values; LDH: lactate dehydrogenase; INR: international normalized ratio; aPTT: activated partial thromboplastin time.

Variables	Value	Reference values
Hemoglobin g/dl	7.5	11-14
MCV fl	92	81-94
PLT 10^3^/mmc	247	140-440
Reticulocytes %	4.2	0.6-2.1
LDH mg/dl	603	135-214
INR	1.1	0.8-1.2
aPTT sec.	26.4	28-40
Fibrinogen mg/dl	608	200-400
Direct Coombs test	Neg	Neg
Haptoglobin mg/dl	113	30-200
Indirect bilirubin mg/dl	0.40	< 0.8
C_3_ mg/dl	84	90-180
C_4_ mg/dl	30	10-40

Taking into account the patient’s medical history and the development of AKI, a diagnosis of likely scleroderma renal crisis was put forward. Waiting for laboratory results, we therefore resorted to the PLASMIC score to predict the probability of severe ADAMTS13 deficiency [8,9]. The score indicated a very low probability of ADAMTS13 activity deficit <10%, as it was later confirmed by the laboratory, thus excluding a possible TTP (Table 3).

**Table 3:** The patient's PLASMIC score Probability of ADAMTS 13 activity under 10%: score 0-4 = 0-4%; 5 = 5-24%; 6-7 = 62-82%. PLASMIC: Platelet count; combined hemoLysis variable; absence of Active cancer; absence of Stem-cell or solid-organ transplant; MCV; INR; Creatinine; INR: international normalized ratio; MCV: mean corpuscular volume

	Variables	Score	Patient
P	Platelet count < 30 x 10^9^/L	(+1)	0
L	HemoLysis variables	(+1)	
	bilirubin – indirect >2.0mg/dL)		
	or reticulocyte count > 2.5%		1
	or undetectable haptoglobin		
A	No Active cancer	(+1)	1
S	No history of Solid-organ or stem-cell transplant	(+1)	1
M	MCV < 90 fl	(+1)	0
I	INR < 1.5	(+1)	1
C	Creatinine < 2.0 mg/dl	(+1)	0
	ADAMTS13 Lab. result	25.7%	4 = 0-4%

**Figure 1:**
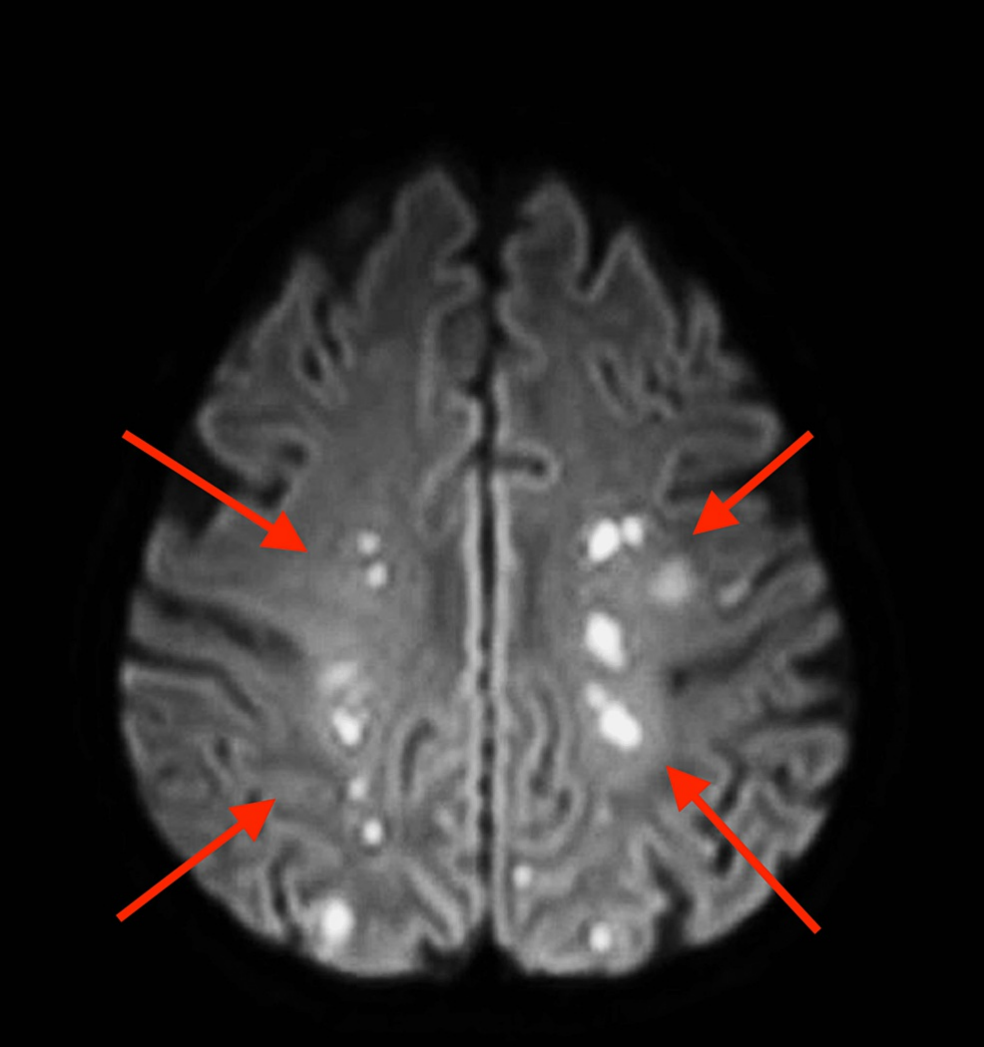
Brain MRI scan showing multiple ischemic lesions Red arrows point to evident ischemic areas spreading in both cerebral hemispheres.

Complement levels were near normal; although hemolytic uremic syndrome (HUS) could not be definitely ruled out, no further investigations were performed. Laboratory data were considered to be evidence of TMA, secondary to diffuse endothelial damage present in scleroderma and severe hypertension [5,10]. Renal ultrasound showed no abnormalities. At chest X-ray, the patient presented signs of interstitial thickening, pulmonary congestion, and bilateral pleural effusion. Treatment was started with an endothelin receptor antagonist (bosentan), converting enzyme inhibitor (captopril), and prostacyclin analog (iloprost), closely monitoring blood pressure. Due to the inadequate reaction to therapy and the supervening oligoanuria in continuing worsening renal damage, hemodialysis was started, remaining dialysis-dependent. The serious conditions prevented us from obtaining a kidney biopsy. A few days later, the patient had progressive dyspnoea, chest pain, and hypotension with a paradoxical pulse. An echocardiogram revealed pericardial effusion with cardiac tamponade requiring pericardiocentesis with pericardial drainage, removing bloody effusion. Acute pancreatitis was diagnosed 10 days later, and confirmed by clinical, laboratory, and instrumental investigations. BP values ranged between 95/60 and 105/70 mmHg. An ischemic stroke occurred with aphasia and right hemiplegia 35 days after admission. Multiple supra- and subtentorial ischemic lesions were evident at MRI (Figure 1).

No right-to-left intracardiac shunt was detected, making an embolic origin of the cerebral lesions highly unlikely. The patient expired five days later.

## Discussion

We described an unusual case of SRC, with a poor clinical course, considering the rarity of its occurrence and the observed complications.

About 40% of SRC show evidence of microangiopathic hemolytic anemia, which is defined by the presence of schistocytes on peripheral blood smear, reticulocytosis; a negative direct Coombs test; increased LDH (tissue ischemia and cell lysis); decrease in haptoglobin levels; indirect hyperbilirubinemia [11]. Microangiopathic hemolytic anemia, resulting from the fragmentation of red blood cells due to partial vascular occlusion by platelet aggregates, and thrombocytopenia (or a >25% decrease in platelet count), resulting from platelet aggregation and consumption, are typically present in TMA. In a small percentage of patients, SRC is normotensive, but with much more severe hemolytic anemia and thrombocytopenia [12].

However, SRC can masquerade as thrombotic thrombocytopenic purpura (TTP), and considering the similarities between the manifestations of SRC and TTP, microangiopathic hemolytic anemia (MAHA), microvascular thrombosis (TMA), and renal failure, these conditions could be mistaken for each another clinically, therefore, delaying proper diagnosis and treatment [4]. TMA includes TTP and HUS. Whereas TTP is related to deficiency of ADAMTS13, HUS is observed in patients developing infection by Shiga toxin-producing enterobacteria and inherited or acquired abnormalities of the alternative complement pathway (atypical HUS). SRC is characterized by severe hypertension with features of TMA and rapidly progressive renal failure related to vascular lesions (fibrointimal sclerosis with lumen narrowing). Considering overlapping syndromes and the various disorders that could be associated with TMA, we used the PLASMIC score in the differential diagnosis. The treatment of SRC is by ACE inhibitor therapy while the early initiation of plasma exchange in other causes of TMA may be life-saving. ACE inhibitors are contraindicated in patients undergoing plasma exchange because of possible severe hypotension. The PLASMIC score provided guidance for making suitable treatment plans, effectively impacting the patient’s prognosis and outcome. The calculated PLASMIC score of this patient, with the information provided, was 4 points (Table 3), which conveyed a very low risk of ADAMTS13 activity deficit, later confirmed by direct laboratory measurement. The PLASMIC score helped rule out TTP, making secondary TMA to SRC the more likely correct diagnosis, without any further delay in the specific treatment.

As far as the cerebral ischemic event that affected the patient was concerned, the PLASMIC score and laboratory results ruled out ADAMTS 13 activity deficit, and, moreover, no right-to-left intracardiac shunt was detected. Considering the large number and dissemination of the lesions, it was likely related to TMA and diffuse endothelial involvement. The cerebral thrombosis and pancreatitis, events that are reported here have been described as extrarenal manifestations of TMA [13]. Furthermore, recent studies have reported a significantly higher incidence of cerebral ischemic events during SRC [3]. A limitation in our approach was the unavailability of renal tissue immunofluorescence pattern, but the progressively worsening clinical conditions did not allow us to safely carry out a renal biopsy. However, with what had been demonstrated by clinical findings, the patient's diagnosis was clear. Studies of renal pathology in scleroderma indicate a preglomerular arteriolar pathology compared to the predominantly glomerular involvement in other forms of TMA [1]. The hypothesis of a complement role in the manifestation of SRC has also been advanced, proposing eculizumab administration to mitigate the clinical effects associated with secondary TMAs, but sound and definite evidence of the efficacy of this treatment seems insufficient to support this indication, at least for the time being [14,15].

Pericardial involvement in scleroderma occurs clinically in 5-16% of cases, and cardiac involvement is generally associated with a poor prognosis [16]. The rapid formation of pericardial effusion with a tamponade effect that occurred in this case may have been due not only to renal failure but also to the reduced elasticity and distensibility of the fibrotic pericardium. Renal damage requiring dialysis occurs in 50-60% of SRC in the acute phase, and half of them remain dialysis-dependent. The need for renal replacement therapy implies a more severe prognosis [7].

In the case here reported, risk factors for developing scleroderma renal crisis were represented by the short duration of the disease (about 4 years), the progression of skin involvement, and treatment with prednisone at doses higher than 15 mg/day within the previous three months [17,18]. As far as autoantibodies are concerned, the presence of anti-topoisomerase I antibodies, as this was the case, seems to be associated with a lower risk of renal crisis than anti-RNA-Polymerase III antibodies, which are associated with a greater risk of developing SRC [19]. In scleroderma, the presence of visceral organ involvement is associated with significantly worse survival, and the greater the number of organs involved, the lower the long-term survival rate [20]. Pulmonary and cardiac involvement had already been documented in the patient described here: renal involvement dramatically worsened the prognosis. Multiple findings, even though rare, including the presence of low-risk antibodies, TMA, and large pericardial effusion, caused the progressive deterioration of the general conditions, with a series of subsequent multiple organ dysfunctions that ultimately caused death.

## Conclusions

Even though SRC is an uncommon emergency, it is associated with high complication rates and in-hospital multiorgan failure. In clinical practice, we describe the case of a severely ill patient with MAHA and TMA secondary to SRC, for whom acute, time-critical decision-making was required. TMA can be associated with significant morbidity and mortality, and its complications present a challenging clinical sequence of events requiring an interdisciplinary course of action. In the present case report, beyond the typical renal involvement, uncommon complications, such as pancreatitis and multiple bilateral ischemic lesions, were regarded as organ damages related to secondary TMA. Scleroderma renal crisis still represents a very serious event with a potentially unfavorable prognosis for both the survival of the kidney and the patient. It is an early need to clinically differentiate risk from other pathology causing rapid renal deterioration since treatment and the related prognosis differ. Recognizing and preventing the potential risk factors associated with its onset are just as fundamental as the prompt therapeutic intervention based above all on the use of ACE inhibitors. Although it is no longer the leading cause of death from scleroderma, it is a major cause of morbidity and mortality. Early treatment and collaboration between rheumatology and renal physicians can improve patient outcomes. The mortality of SRC is still high, and research on causes precipitating and exacerbating SRC is warranted for developing effective therapies.
